# The forgotten airway: central airway pathology in the ICU

**DOI:** 10.3389/fmed.2025.1712110

**Published:** 2026-01-12

**Authors:** Alessandro Marchioni, Antonio Moretti, Luca Tabbi', Francesco Livrieri, Martina Tartaglia, Michele Gentile, Alberto Scorsone, Francesco Mattioli, Roberto Tonelli, Enrico M. Clini

**Affiliations:** 1 Respiratory Disease Unit, Department of Surgical and Medical Sciences of Motherhood and Child, Azienda Ospedaliero-Universitaria di Modena, Modena, Italy; 2 Department of Otolaryngology-Head and Neck Surgery, Azienda Ospedaliero-Universitaria di Modena, Modena, Italy

**Keywords:** central airway obstruction, expiratory dynamic airway collapse, ICU, mechanical ventilation, obstructive fibrinous tracheal pseudomembrane, tracheal laceration, tracheal resection, tracheal stenosis

## Abstract

Central airway diseases, though infrequent, represent a critical yet underrecognized cause of respiratory failure in the intensive care unit (ICU). These disorders encompass a wide range of pathologies—including central airway obstruction (CAO), tracheal stenosis, tracheomalacia, tracheomegaly, tracheoesophageal fistulas, and post-intubation lacerations—which can significantly complicate ventilation, extubation, and weaning. This narrative review explores the complex anatomical and physiological properties of the trachea and main bronchi, highlighting the profound impact of their even partial occlusion on airway resistance and work of breathing. We present the pathophysiological bases, diagnostic strategies, and up-to-date management options for both benign and malignant CAO, with particular attention to the ICU setting where urgent interventions are often required. The review also addresses iatrogenic airway injuries related to mechanical ventilation and airway equipment, analyzing both conservative and interventional therapeutic approaches, including endoscopic techniques and airway stenting. Special focus is given to the implications of central airway pathology in weaning failure, emphasizing the importance of early recognition to avoid unnecessary reintubations. Overall, this work aims to raise awareness among intensivists and pulmonologists of the pivotal role that central airway diseases may play in respiratory deterioration, and to advocate for a structured, multidisciplinary approach to their prompt diagnosis and treatment.

## 1 Background

Central airways, consisting of trachea and mainstem bronchi, play a critical role in gas conduction during tidal ventilation as well as in ensuring tracheal and bronchial secretion drainage. The trachea is a tube-shaped organ, averaging 11.8 cm in length (range: 10–13 cm), composed of 16–20 U-shaped cartilage rings that support a membranous posterior wall (the trachealis muscle). This heterogeneous structure is also found in the main stem bronchi and the extra-pulmonary portions of the lobar bronchi (1). Given this unique architecture, pressure changes from spontaneous ventilation influence central airway behavior by altering transluminal pressure, which depends on the balance between intra-luminal and extra-luminal pressures. The airflow is critically dependent on the inner diameter of the trachea, being the resistance to flow inversely proportional to the radius according to the law of Hagen-Poiseuille (2). Thus, during spontaneous breathing, central airways diseases such as tracheal stricture, malacia or functional collapse, increase work of breathing by reducing the airways' conductive capacity. Therefore, central airway obstruction from various diseases can lead to acute respiratory failure, often requiring immediate intervention. The aim of this narrative review is to present the state-of-the-art on central airway diseases requiring ICU management, focusing on physio-pathological features and clinical implications of these underestimated diseases.

## 2 Methods

This narrative review was conducted following a structured but non-systematic approach to identify the most relevant literature on central airway diseases in critically ill patients. A comprehensive search was performed in MEDLINE from January 1990 to September 2025. The following keywords and their combinations were used: “*central airway obstruction,” “tracheal stenosis,” “tracheal laceration,” “tracheoesophageal fistula,” “tracheomalacia,” “expiratory dynamic airway collapse,” “ICU,” “mechanical ventilation,” “weaning failure,” “tracheobronchial injury,” “tracheal resection,” “tracheomegaly,” and “Obstructive Fibrinous Tracheal Pseudomembrane.”* We included original articles, clinical studies, case series, guidelines, and review papers that focused on the epidemiology, pathophysiology, diagnosis, and management of central airway pathology in the intensive care setting. Papers were excluded if they were not written in English, did not involve human subjects, or addressed exclusively pediatric congenital airway diseases without implications for adult ICU practice. Given the heterogeneous nature of the available evidence, no formal quality assessment or meta-analysis was performed. Reference lists of key articles and recent guidelines were additionally screened to identify further relevant studies. The final selection aimed to emphasize both consolidated knowledge and recent advances from the last 5 years.

All radiological and bronchoscopic images included in this review derive from the authors' institutional clinical archive. All patients provided written informed consent for the use of anonymized images for scientific and educational purposes, in accordance with institutional policies and the Declaration of Helsinki.

## 3 Trachea behaviors during spontaneous breathing

The trachea is subjected to a variable transmural pressure during spontaneous breathing according to its different segments. Indeed, the distal two-thirds of the trachea are intrathoracic, where extraluminal pressure is equivalent to pleural pressure (P_pl_), while the proximal third of the trachea is extra thoracic, where extraluminal pressure corresponds to atmospheric pressure (P_atm_). Therefore, the cervical and intrathoracic portions of the trachea are subjected to different external and transmural pressures. The transitional area is at the cervicothoracic junction, opposite the suprasternal notch, and is subjected to unique pressure dynamics differing from either neck or thorax (3). At the same time, the trachea moves with cervical extension and flexion modifying the location of the extrathoracic compartment. The trachea slides considerably between compartments during respiration further affecting the complex mechanical forces acting on the organ (4). Breathing is driven by pressure gradient between the alveolar pressure and atmospheric pressure at the airway opening. During inspiration diaphragm contractions decrease P_pl_ generating a pressure gradient along the tracheal bronchial tree from P_atm_ at the mouth to the alveoli. In this process the pressure outside the intrathoracic portion of the airways is more negative than the intraluminal central airways pressure. Therefore, the resulting transmural pressure causes dilatation of the intrathoracic airways. In the extrathoracic portion of the trachea, the collapse of the organ exposed to a positive transmural pressure is prevented by the rigidity of its wall and stiffening of pharyngeal muscles by neurological impulses. During exhalation, alveolar pressure (P_alv_) exceeds P_pl_ by an amount equal to recoil pressure of the lung (PL), thus driving air out, and the intraluminal airway pressure becomes positive in both intrathoracic and extrathoracic portion of the trachea (5). With forced exhalation the driving pressure which leads air outflow is expressed by the equation:


$$\begin{array}{l} P_{alv}\;=\;P_{pl}\;+\;P_{L} \end{array}$$ (1)

Driving pressure changes during forced compared to quiet exhalation, being P_L_ and P_pl_ both positive in sign, while in quiet expiration P_pl_ remains negative and pressure generated to driving air out is determined by P_L_ only. As a pressure drop between the alveoli and atmosphere occurs along the airways when expiratory flow begins, it follows that during forced exhalation the pressure drop from the alveoli to some point within the airway must equal P_pl_. At this point the intraluminal airway pressure equals P_pl_, generating a physiological consequence named *equal pressure point* (EPP), which results in dynamic compression of the airways in the thoracic portion. In the upstream segment (between alveoli and EPP) the pressure inside the airways exceeds P_pl_ dilating the airways, while in the downstream segment (between EPP and thoracic outlet) intra-airway pressure is below P_pl_ and the airways are compressed. However, as the extrathoracic portion of the trachea is subjected to P_atm_ from the outside, the slight increase of intraluminal pressure consequent to forced exhalation results in a moderate dilatation of the airway in this compartment (6). In the course of a forced expiratory maneuver the EPP is found in the intrathoracic trachea immediately after the early peak flow, and subsequently it moves toward the lung periphery. The ability of tracheal tissue to deform radially and the mechanical proprieties of the central airways play a key role in maintaining airway stabilization during breathing. Tracheal rings, composed of hyaline cartilage, can avoid complete tracheal collapse despite changes in the transmural pressure (7). Membranous posterior wall contributes most significantly to the changes in cross-sectional area, despite the trachealis muscle contraction acts as adjustment of the trachea diameter (8). Central airway pathology, such as tracheomalacia or benign tracheal stenosis, modifies the physiological behavior of trachea during breathing making airway management difficult in the ICU.

## 4 Respiratory failure due to central airway obstruction

**Figure 1:**
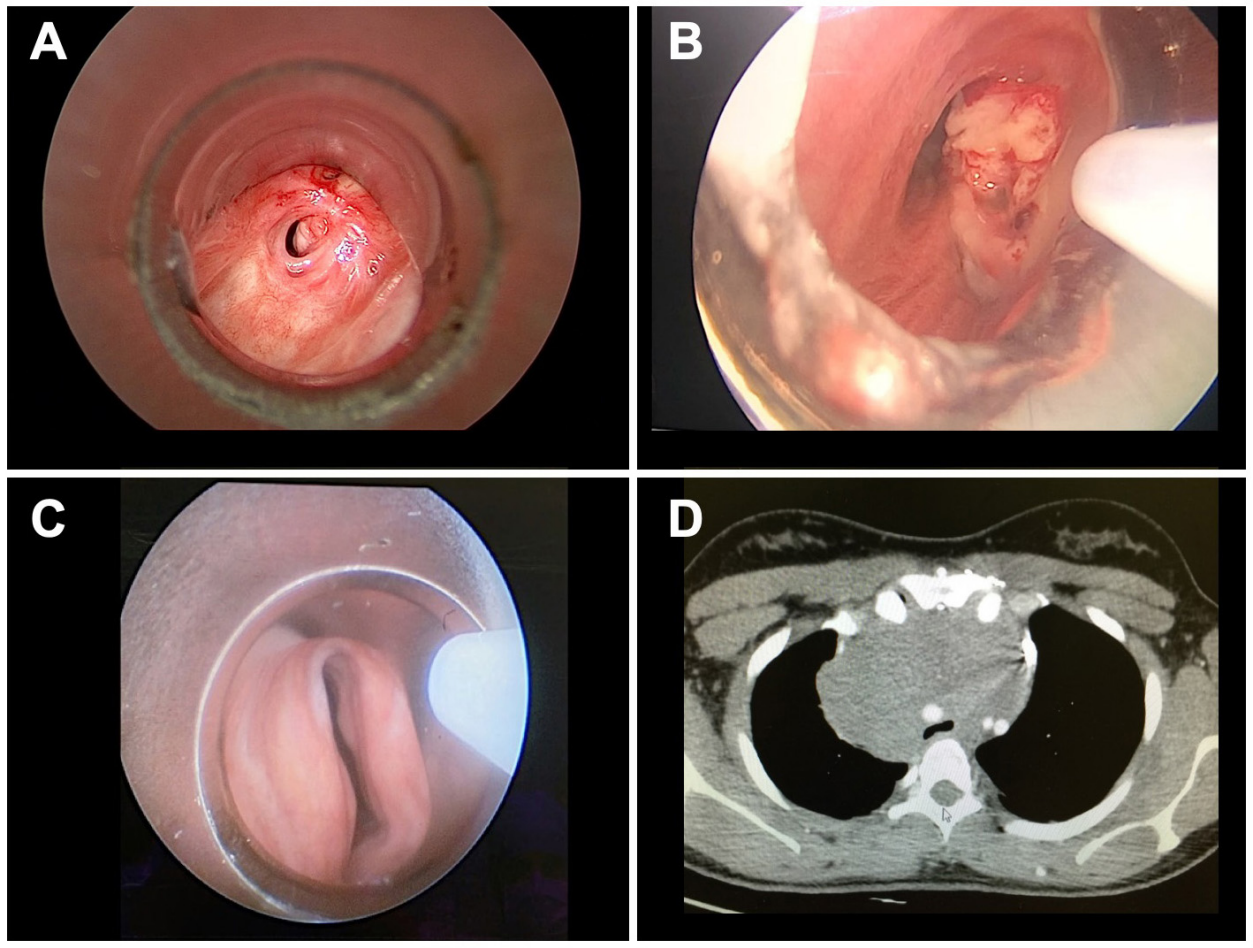
CAO classification: based on the nature of the obstruction, CAOs are classified as benign or malignant. Anatomically, CAO can be classified into three distinct categories: intrinsic, extrinsic, and mixed. **(A)** Benign CAO due to tracheal stenosis. **(B)** Malignant CAO due to squamous cell carcinoma with carinal invasion. **(C)** Benign CAO due to saber-sheath malacic trachea. **(D)** Malignant CAO due to extrinsic compression by mediastinal lymphoma.

Central airway obstruction (CAO) is generally defined as airflow limitation due to > 50% occlusion of the trachea, main-stem bronchi, bronchus intermedius, or a lobar bronchus (9). Several diseases, both benign and malignant, can bring to CAO. Benign strictures, such as post-intubation, post tracheostomy or idiopathic tracheal/subglottic stenosis, comprise the majority of the CAOs. Malignant CAOs are generally the result of intraluminal growth of a primitive tracheal tumor or a bronchogenic cancer. However, malignant CAO can also be the result of compression of the airways by tumors of surrounding organs such as esophagus, thyroid, mediastinal lymphomas or other malignancies (10). Anatomically, CAO can be classified into three distinct categories: intrinsic (airway occlusion due to lesions inside the airways), extrinsic (airway stenosis due to extrinsic compression), and mixed (Figure 1). Another cause of CAO is aspiration of foreign bodies, mainly occurring in children, which may result in acute respiratory failure. Patients with CAO often present with dyspnea, orthopnea, cough and wheezing. Not infrequently, especially when airways occlusion occurs progressively over time, airway obstruction is confused with asthma or chronic obstructive pulmonary disease (COPD) exacerbation. The presence of stridor, inspiratory wheezing, and the change of voice features, should all raise suspicion of CAO even if chest X-ray excludes obvious lesions. Tracheal and carinal lesions are often not identifiable on chest radiograph, and require further investigation such as chest CT scan, or flexible bronchoscopy. In case of foreign body inhalation, symptoms can vary considerably according to the site of the foreign body in the airways. Larynx or tracheal occlusion causes immediate respiratory distress or stridor, while if the foreign body migrates to the bronchi clinical signs are much less constant and nonspecific. Radiological hallmark of an aspirated foreign body in children is the hyperinflation of the affected lung due to the check valve mechanisms (i.e., air enters the bronchus around the foreign body but cannot exit). In stable patients, chest X-ray should be performed during expiration to emphasize the difference between the lungs. However, obstructive emphysema with air trapping is detected only in 17%−69% of cases, and forced respiratory radiographs cannot always be obtained in young children, thus requiring further investigations. During the acute phase, chest radiograph has low sensitivity and specificity for the diagnosis of bronchial foreign body and is reported as normal in 14%−37% of cases (11).

Besides foreign bodies aspiration, congenital and acquired pediatric disorders such as tracheal or subglottic stenosis, CAO secondary to intraluminal masses or extrinsic compression, TBM and TEF, may benefit from treatments performed in rigid bronchoscopy.

Table 1 summarizes the causes of CAO.

**Table 1:** Causes of central airway obstruction (9, 10).

**Category**	**Cause**	**Notes**
Benign—iatrogenic	Post-intubation stenosis	Most common benign cause; ischemic injury from cuff pressure leading to fibrosis
	Post-tracheostomy stenosis	Scarring and granulation tissue at stoma site
Benign—inflammatory/autoimmune	Granulomatosis with polyangiitis	Necrotizing granulomas causing subglottic or tracheal stenosis
	Relapsing polychondritis	Inflammatory destruction of airway cartilage with collapse
	Sarcoidosis	Granulomatous infiltration and airway narrowing
Benign—idiopathic	Idiopathic subglottic stenosis	Predominantly affects middle-aged women; abnormal wound healing response
Benign—infectious	Tuberculosis	Cicatricial scarring and bronchial stenosis
	Fungal infections (e.g., histoplasmosis)	Granulomatous strictures or extrinsic compression from lymphadenopathy
Benign—other	Inhalation injury (thermal, chemical)	Fibrosis and cicatricial narrowing
	Benign tumors (papilloma, hamartoma, lipoma, fibroma)	Mechanical obstruction
Malignant—primary airway tumors	Squamous cell carcinoma	Most common primary tracheal malignancy
	Adenoid cystic carcinoma	Slow-growing but infiltrative along airway wall
	Carcinoid tumor	Endoluminal growth obstructing bronchus or trachea
Malignant—secondary/extrinsic	Lung cancer (NSCLC, SCLC)	Direct invasion or extrinsic compression
	Thyroid carcinoma	Tracheal compression or invasion
	Esophageal carcinoma	Posterior tracheal wall invasion
	Lymphoma/mediastinal tumors	Bulky adenopathy compressing airways

### 4.1 Physiology of CAO

For physiological reasons both benign and malignant CAOs become clinically evident only when airway lumen is significantly occluded, making airway management a challenge for the physician. Airway resistance (R) due to tracheal stricture seems to obey the *Hagen-Poiseuille* equation, which defines the relationship between airway radius and airflow resistance through the following equation:


$$\begin{array}{l} R=\frac{8\text{μL}}{\pi d^{4}} \end{array}$$

Where *R* is resistance, μ is fluid viscosity, *d* is the tube diameter, and *L* is the tube length. Thus, the airway resistance being inversely proportional to the second power of the airspace cross-sectional area (A).


$$\begin{array}{l} R\propto A^{-2} \end{array}$$

Based on this assumption, a 50% reduction of the cross-sectional airway area results in a 16-fold increase in airflow resistance. However, Hagen-Poiseuille equation is valid only for laminar flow in straight circular tubes, while it is less reliable when looking at the upper airways and subglottic area, where airflow is near the transition from laminar to turbulent flow. In the upper airways, the Bernoulli obstruction theory better describes the relationship between airway resistance and cross-sectional area, which results as in the following proportion:


$$\begin{array}{l} R\propto A^{-1} \end{array}$$

**Figure 2:**
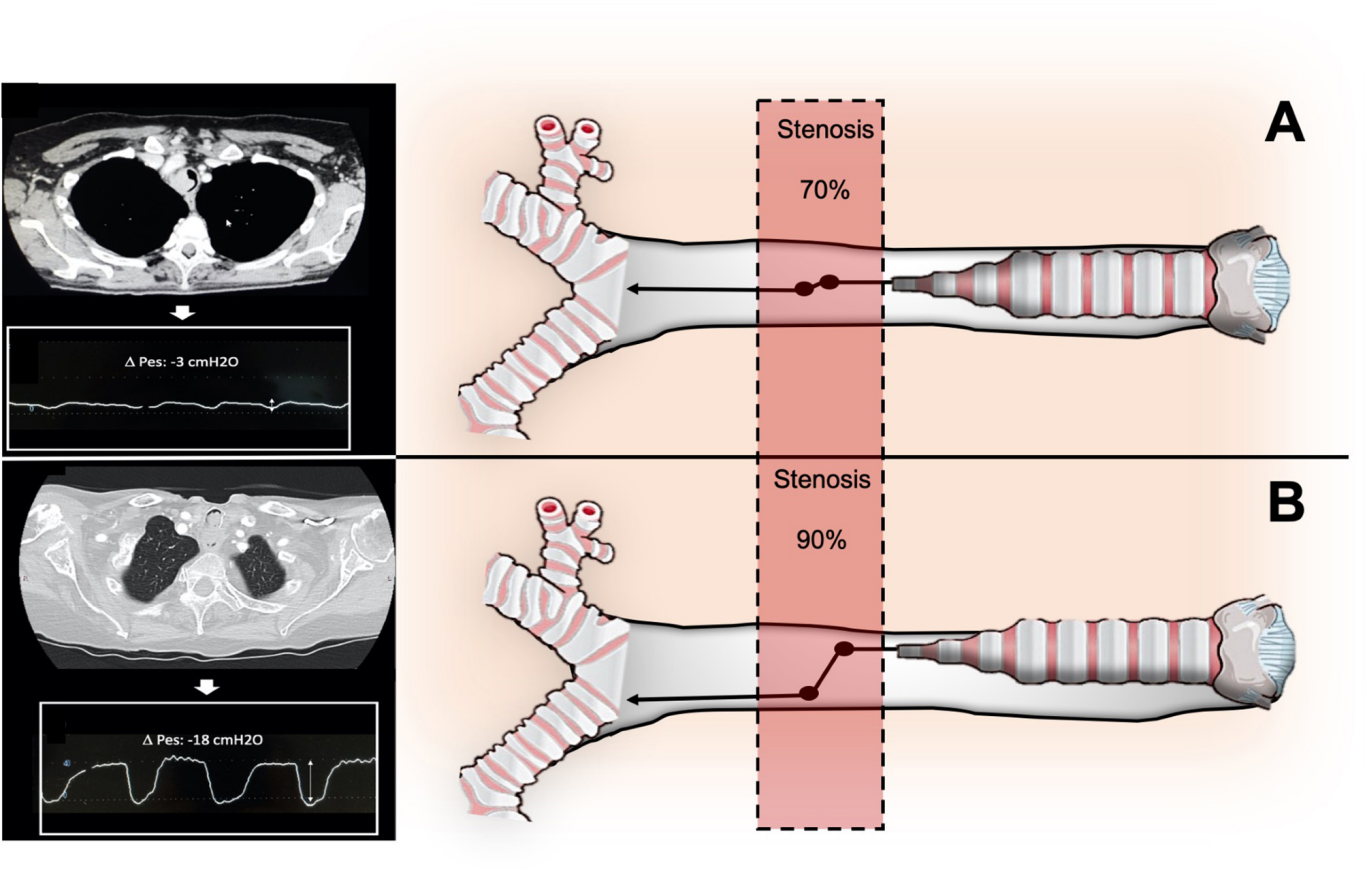
Relationship between degree of stenosis (**A**: stenosis < 70%; **B**>70%), airway pressure drops across the stenosis, and inspiratory effort (DPes) in CAO.

Therefore, in the upper airway, a cross-sectional area reduction has less impact on resistance, as the Bernoulli obstruction theory predicts that a four-fold reduction in cross-sectional area would result in a four-fold increase in upper airway resistance (12). Despite airway cross-sectional area is a critical factor for airway resistance, it is also a key factor in determining the pressure variation along the stenotic airway, which consists of a significant drop at the obstruction level. The pressure variation along the stenotic airway is the main determinant of the patient's work of breathing (WOB), which becomes significant only when the stenosis is severe (Figure 2). Indeed, the pressure reduction at the stricture level dramatically increases only when more than 70% of the tracheal lumen is obliterated, and increases proportionally with the increase of airflow as described by the following equation:


$$\begin{array}{l} \Delta P_{\left[stenosis\right]}^{loss}=\rho \text{K}\frac{Q^{2}}{2A^{2}} \end{array}$$

Where *Q* is bulk flow, *A* is cross-section of the airway, *p* is gas density, and *K* is an empirically determined constant for any specific geometrical feature (13). The equation above well describes how the respiratory symptoms, due to the pressure drop through the stenosis, arise only at an advanced stage of tracheal stenosis or in conditions of increased airway flow as during physical exertion. Therefore, usually patients suffering from CAO come to the attention of the physician when tracheal occlusion is critical and the inspiratory effort i.e., pleural pressure variation, or Delta esophageal pressure (ΔPes) generated to overcome the airway resistance is considerable.

ΔPes refers to the change in esophageal pressure (PES) over a specific phase of the respiratory cycle, typically measured as the difference between end-inspiratory and end-expiratory esophageal pressures during tidal breathing. In the context of intrapleural pressure measurement, ΔPes is used as a surrogate for the change in pleural pressure (ΔPpl), since esophageal pressure closely tracks pleural pressure swings, even though absolute values may differ due to anatomical and physiological factors (14).

ΔPes is commonly used to estimate the pressure generated by the respiratory muscles and to assess chest wall and lung mechanics. The measurement is performed using an esophageal balloon catheter, and the value is calculated as:


$$\begin{array}{l} \Delta PES=PES_{inspiration}-PES_{expiration} \end{array}$$

## 5 Management of CAO

CAO is a life-threatening disorder associated with poor prognosis.

Recent guidelines (2019–2024) from ACCP and WABIP have substantially refined the diagnostic and therapeutic framework for malignant and benign CAO, particularly regarding stent selection, indications, and peri-procedural management (15, 16).

In the management of CAO, the first step is the identification of the underlying disease, as the presenting symptoms—such as wheezing, cough, and dyspnea—may closely resemble COPD and asthma, particularly in patients with undiagnosed malignancies or iatrogenic/idiopathic benign tracheal stenosis. The second step is to guarantee adequate alveolar ventilation to the critically ill patient. Finally, the third step is to allow definitive surgical or endoscopic treatment. CAO, being a heterogeneous disease, manifests with different clinical scenarios: (1) asphyxia or severe acute respiratory failure; (2) dyspnea with exercise limitation. In patients with suspected severe CAO with life-threatening presentation, diagnosis and treatment should not be delayed, and guaranteed alveolar ventilation becomes a priority. Identifying the obstruction site is the key to deciding the best airway management.

Over the past 5 years, flow-controlled ventilation (FCV) and ultra-thin cuffed tubes (e.g., Tritube^®^) have emerged as transformative tools for managing complex upper airway obstructions, allowing stable ventilation while minimizing air-trapping and barotrauma (17, 18).

Supraglottic or subglottic obstruction should be treated with tracheotomy as soon as possible. However, supraglottic airway devices (SADs) may allow ventilation in patients with severe subglottic or tracheal stenosis, where tracheal intubation is difficult. Successful management with the use of SADs, especially using the laryngeal mask, in patients suffering from CAO has been widely described in the literature (19–21). If mask ventilation is attempted, a slower rate with longer respiratory and expiratory times is required to allow for adequate tidal exchange and to avoid air trapping. If hypotension develops after the initiation of positive pressure ventilation, differential diagnosed included pneumothorax due to air trapping and obstructed exhalation (22). Flexible bronchoscopy can help to define CAO causes and to decide the best options for airway management and anesthesia. The fixed vs. dynamic characteristics of the stenosis can help to tailor anesthesia induction and ventilation strategy to the patient's lesion. Usually, the use of neuromuscular blockade is avoided, preferring to maintain spontaneous breathing during assisted ventilation. However, when the predominant component of the stenosis is dynamic airway collapse during inspiration, due to tracheal malacia or excessive airway collapse, the best airway management may be achieved by inhibition of spontaneous breathing and application of positive pressure through a tracheal tube or SADs. In these cases, the use of neuromuscular blockers and controlled mechanical ventilation may be the best strategy to manage the airways. If the CAO is fixed the approach may be different and depends on the possibility of overcome the stenosis to ensure ventilation. For mid-tracheal stenosis, tracheal tube can be placed, under bronchoscopic guidance, cephalad to the stricture. Alternatively, the stricture can be passed with small caliber tubes (6 mm or less). In more complex conditions, veno-arterial extracorporeal membrane oxygenation (ECMO) may be considered to achieve adequate gas exchange while awaiting definitive treatment. In less critical conditions, alveolar ventilation can be guaranteed with more sophisticated methods, to allow tracheal or laryngeal surgery. In these scenarios, airway management can be achieved by High Frequency Jet Ventilation (HFJV), a technique based on a non-cuffed narrow-bore endotracheal tube with outer diameter of around 4.3 mm, which can be positioned downstream of the stenosis, and a high frequency ventilator which insufflates small volumes of air at a supra-physiological rate (100–150/min). However, this technique requires high pressure to develop air flow, exposes the patient to the risk of barotrauma and hyperinflation due to air-trapping, especially if the diameter of the trachea proximal to the tracheal catheter tip is too small to permit complete exhalation of the tidal volume around the tracheal catheter (23). Recently, “flow-controlled” ventilators (FCVs) were developed for difficult airway management as they are able to actively remove air out of the lungs using negative pressure through small diameter cuffed tubes (e.g., Tritube, OD of only 4.4 mm, < 3 mm inner diameter), thus avoiding the air-trapping phenomenon (24). The negative pressure used to aspirate air out of the lungs, is generated applying Bernoulli's principle, forcing a current of air through a small tube inside the ventilator. FCVs ventilators seems very promising in difficult airway management allowing stabilization of ventilation, while awaiting definitive endoscopic or surgical treatment.

### 5.1 Endoscopic treatment of CAO

**Figure 3:**
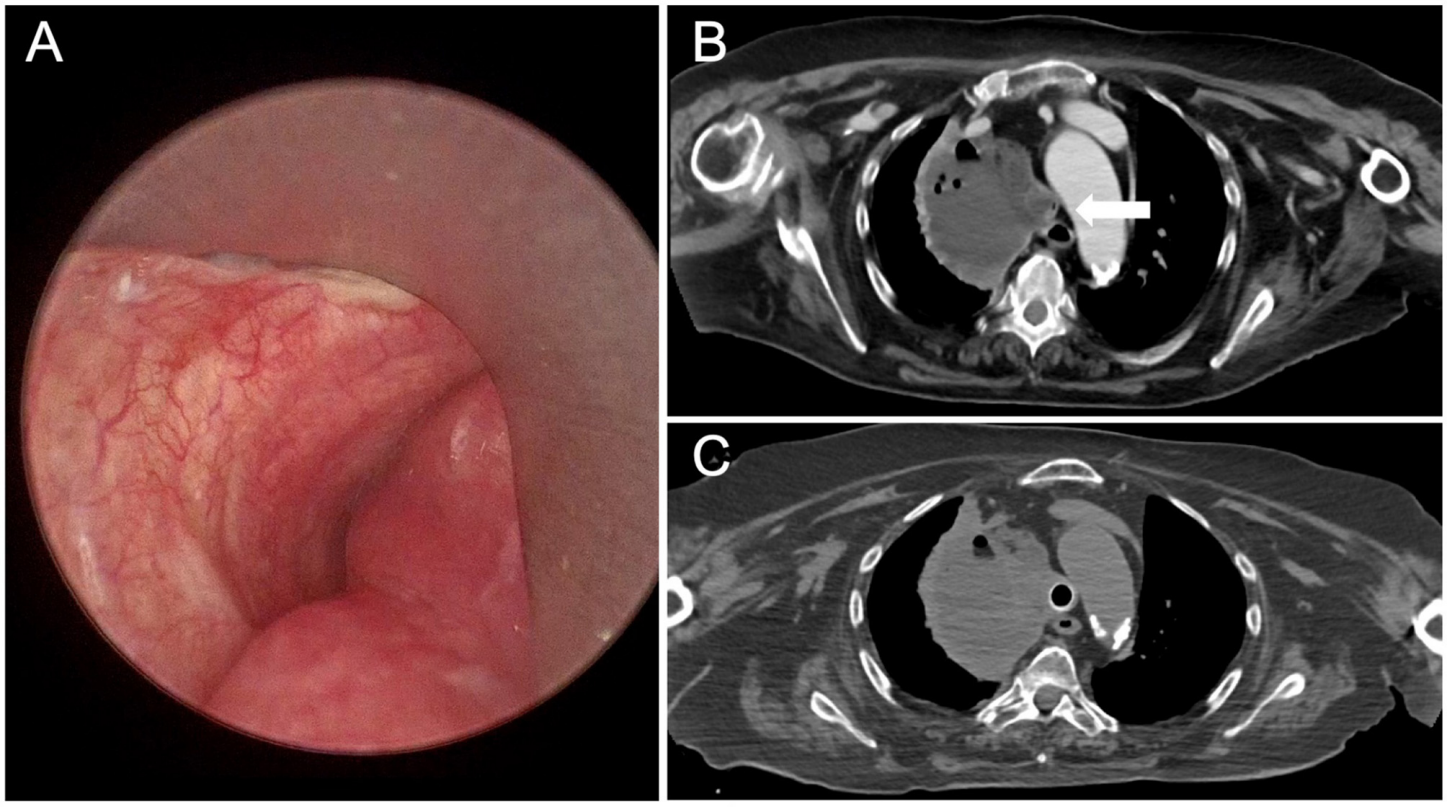
Benign CAO in patient with lung abscess and tracheal compression. **(A)** Bronchoscopic view. **(B)** Chest CT scan showing the axial plan of a trachea with severe stenosis. **(C)** Chest CT axial scan after rigid bronchoscopy, tracheal dilation and stent placement.

The endoscopic approach is the gold standard in case of removal of foreign bodies or in malignant CAO. As outlined in the latest guidelines from the American College of Chest Physicians (ACCP), rigid bronchoscopy plays a pivotal role in CAO due to its rapid and effective ability in airway stabilization (15). It provides a route for ventilation and oxygenation while also offering important therapeutic interventions, such as the mechanical removal of large endoscopic lesions, better control of endoluminal bleeding through high suction and tamponade capacity, and it facilitates the insertion of additional instruments, such as a flexible bronchoscope, rigid forceps, laser, or the placement of stents. The classification of malignant CAOs based on the type of obstruction (i.e., extrinsic compression of the airway or endoluminal occlusion) affects bronchoscopic management, including the decision to stent (16). Airway stent is usually indicated if there is a significant airway compression or after debulking of endobronchial lesion in case of residual stenosis. Rigid bronchoscopy and stent placement should be considered in patients with respiratory failure requiring mechanical ventilation due to malignant CAO. Despite the small body of published data, some studies suggest that airway stenting is an effective intervention to liberate patients from mechanical ventilation. A study by Oki et al. included 30 patients with malignant CAO who required emergency intubation and then underwent therapeutic bronchoscopy and stent placement. Extubation within 48 h after stenting was possible in 93% of patients (28 of 30) (25). Other smaller studies reported success rates in liberating from mechanical ventilation within 24 h ranging from 50 to 100% (26, 27). Furthermore, rigid bronchoscopy may have a role also in the treatment of benign tracheal stenosis, through laser resection and dilatation in simple tracheal stenosis, and stent placement in selected cases of complex tracheal stenosis (28). Figure 3 illustrates stent placement in severe tracheal stenosis due to lung infection.

### 5.2 Surgical treatment of CAO

Open surgical treatment is indicated in a minority of patients suffering from severe CAO, especially in life-threatening diseases and in malignant occlusion of the airway, while tracheal sleeve resection remains the gold standard in benign complex tracheal stenosis (lesions >1 cm in length, with cartilage involvement or malacia). However, in patients who are not suitable candidates for surgery, stent placement achieves a success rate of over 70% (29). In patients suffering from CAO in the subglottic area, a surgical airway should be considered in life-threatening conditions due to difficult airway management. Cricothyroidotomy or tracheostomy can be safely performed in the awake, spontaneous ventilating patient.

## 6 Iatrogenic airway injury in ICU

Airway management through endotracheal intubation or tracheostomy and prolonged invasive mechanical ventilation, can lead to a range of complications affecting the airways, and the trachea in particular. Tracheal stenosis, together with tracheal laceration, tracheomegaly, tracheo-esophageal fistula and tracheo-innominate artery fistula are all life-threatening complications, associated with high rates of morbidity and mortality.

### 6.1 Iatrogenic tracheal and subglottic stenosis

**Figure 4:**
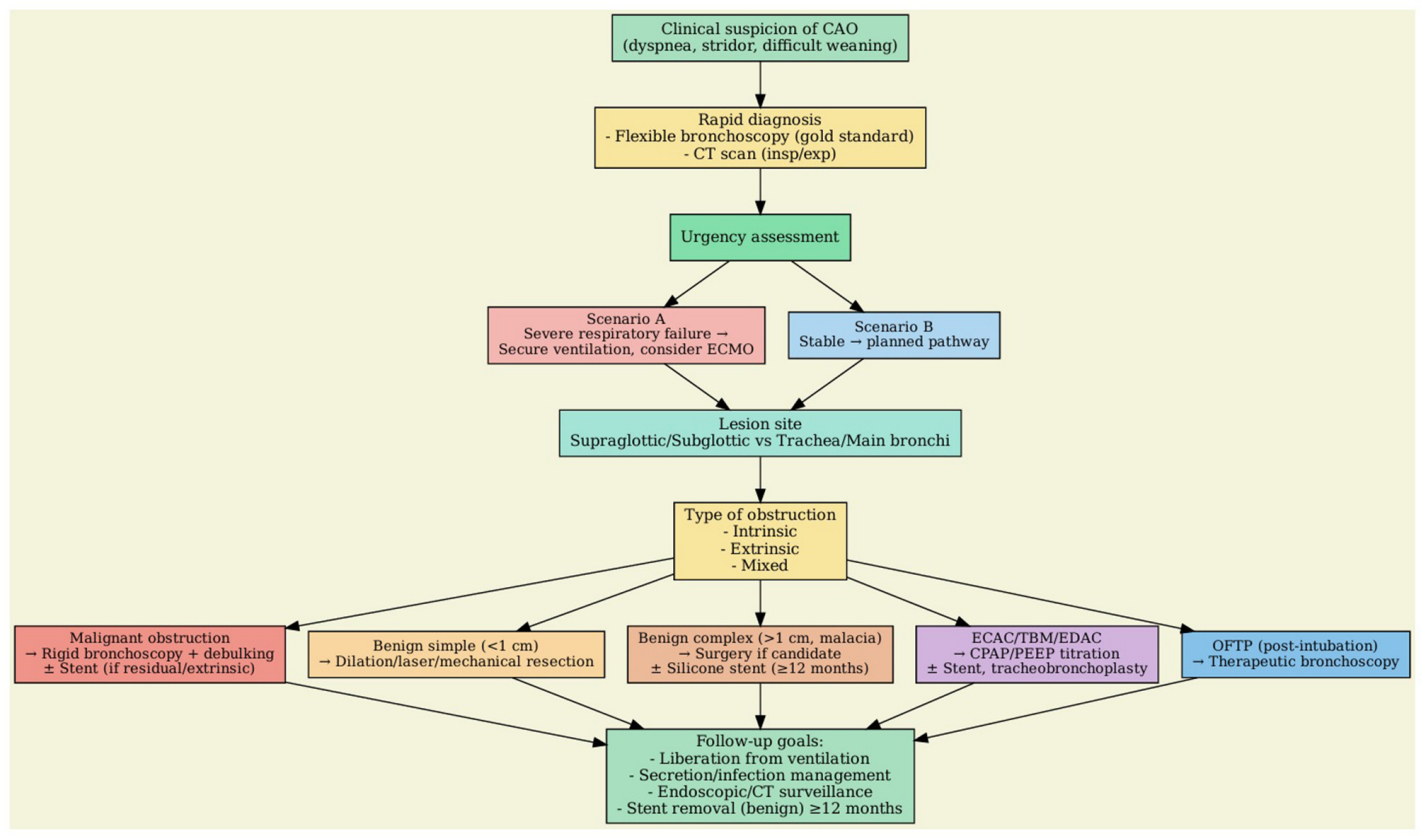
Decision-making algorithm in CAO.

Despite over 50% of patients undergoing endotracheal intubation and prolonged mechanical ventilation experience some degree of laryngeal and tracheal injury, the incidence of significant iatrogenic laryngo-tracheal stenosis (iLTS) is variable, ranging from 6 to 21% (30). In a prospective cohort study that enrolled one hundred patients intubated for more than 12 h, endotracheal tube greater than size 7.0, diabetes, and large body habitus were all predisposing factors for upper airway injury (31). However, the main known risk factor for developing airway stenosis is a prolonged duration of intubation, particularly if >7 days (32). Most cases of post-intubation tracheal stenosis occur due to over-inflation of the endotracheal tube cuff, with pressure higher than 20 cmH_2_O, leading to ischemic injury of the tracheal mucosa, necrosis, to the formation of scar tissue and subsequent stenosis. Post-tracheostomy tracheal stenosis occurs similarly, due to ischemic injury of the mucosa associated with excessive healing, but is usually also associated with cartilage damage. Iatrogenic tracheal stenosis is no longer considered a disease due to an inert scar development in the airway but rather is looked at as a disease due to an impaired immune response and aberrant repair, similarly to other fibrosing diseases. Recent advancement in understanding the mechanisms behind iLTS suggest that the excessive fibrotic deposition is due to various factors such as immune system dysregulation, changes in the extracellular matrix, metabolic alterations, and the role of microbiota (33). Excessive laryngeal-tracheal scarring is sustained by TH2 responses after an airway injury that promote fibroblasts activation and alternative polarization of macrophages to a profibrotic M2 phenotype, leading to aberrant healing. Other factors may contribute to hypertrophic scar formation in critically ill patients such as prolonged hypoxia, and mechanical stress with activation of mechanotransduction pathways (13). Stenoses can generally be divided into two categories: simple and complex. Simple refers to web-like, concentric, membranous stenoses that are shorter than 1 cm and without cartilage involvement. Complex stenoses are characterized by cartilage damage or tracheal malacia, with longitudinal involvement typically longer than 1 cm. Simple stenosis is generally treated with endoscopic scar resection (laser, cold or electric knife) and dilatation with balloon or rigid bronchoscope. Despite recurrence and the need for re-treatment are frequent, prognosis is usually favorable (34). Management of complex stenosis is challenging and surgery remains the cornerstone of treatment. However, in patients unsuitable for surgery, stent placement is more effective than the endoscopic dilatation with balloon in achieving stabilization of tracheal patency (28). Silicone stents are currently the gold standard in the treatment of benign tracheal stenosis and should be considered as a temporary measure during remodeling and stabilization of the airway. The patency of airway after stent removal is achieved in about 40%−75% of cases, depending on the studies (35). However, a critical factor in obtaining effective treatment is how long the tracheal stent is kept in place. Indeed, the stabilization and remodeling process of the airways requires at least 6–12 months, and early stent removal may predispose to stenosis recurrence. Based on the available evidence, silicone stent should be maintained in place for a minimum of 12 months (36). Some studies have evaluated the efficacy of fully covered self-expandable metallic stents (SEMS) in the treatment of benign tracheal stenosis. A study analyzed 30 patients affected by benign tracheal stenosis treated with SEMS placement, showing a clinical success rate of 40%. However, 50% of the stents were subsequently removed due to stent-related complications (37). Further studies have reported a higher complications rate using SEMS compared to silicone stent. Therefore, SEMS should not be considered as the first stent choice in the treatment of inoperable benign tracheal stenosis (38). Figure 4 summarizes the flowchart algorithm for CAO management.

### 6.2 Tracheomegaly

**Figure 5:**
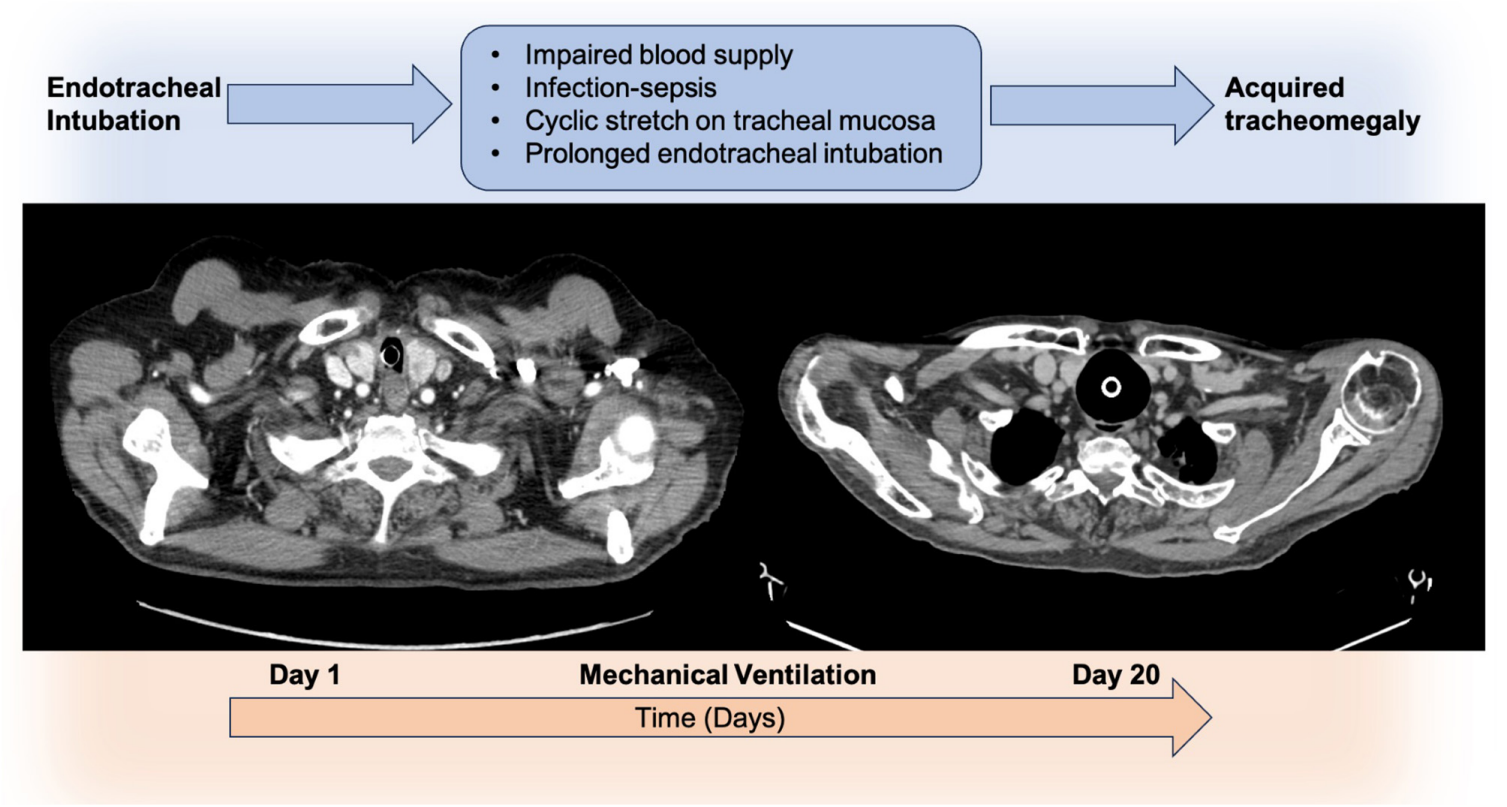
The figure illustrates the axial CT scans of a patient with acquired tracheomegaly after mechanical ventilation. On the **left**: trachea with regular caliber after tracheal intubation and mechanical ventilation. On the **right**: development of tracheomegaly after prolonged mechanical ventilation.

Tracheomegaly is a rare but clinically impactful condition confined to central airways, which can be congenital (named Mounier-Kuhn Syndrome) associated with elastic fiber and smooth muscle atrophy, but also acquired in adulthood (39). Tracheomegaly occurs primarily in patient suffering from Ehlers-Danlos syndrome, cystic fibrosis, pulmonary fibrosis and pulmonary emphysema. However, mechanical ventilation can rarely be the cause of an acquired tracheomegaly (40). In these cases, impaired blood supply in the airway due to pressure-related wall necrosis, infection and cyclic stretch on tracheal mucosa during prolonged endotracheal intubation, may all play a role in the pathogenesis of this disease (Figure 5). Tracheomegaly is diagnosed by radiological evaluation. On CT scan diagnosis is confirmed when the transverse diameter of the trachea exceeds 2.5 cm in men and 2.1 cm in women. Bronchoscopy findings associated with tracheomegaly may be tracheal malacia, narrowing on forced expiration, tracheal diverticula and mucosal inflammation (41). Physiologically, the enlargement of the airway diameter results in increased inspiratory flow speed and propensity to turbulent flow with lower flow rates. Furthermore, airway narrowing during expiration and complete obliteration in the course of forced expiration cause air trapping, and predispose to formation of diverticula between cartilaginous rings. Clinically, manifestations are variable and not infrequently this condition can stay asymptomatic. However, some patients may present ineffective cough, dyspnea, secretions retention and recurrent infections. Acquired tracheomegaly represents a significant management challenge in critically ill patients, particularly within the ICU setting. Tracheomegaly predisposes patients to a spectrum of airway-related complications that can undermine both ventilation strategies and overall respiratory stability. A critical risk is the peritubal cuff leak observed in endotracheal or tracheostomy tubes (42).

The mismatch between the airway diameter and the artificial airway device often needs excessive cuff inflation to achieve adequate tidal volumes and to minimize air leak. However, cuff hyperinflation carries its own risks, including iatrogenic tracheal injury, further dilation, and the potential development of tracheoesophageal fistula, a life-threatening complication in mechanically ventilated patients (43). In addition, persistent cuff leaks may result in aspiration of oral or gastric contents, tidal volume mismatch, and hypercapnia leading to worsening respiratory acidosis. The combination of these factors not only complicates ventilation management but also increases the likelihood of ventilator-associated pneumonia and prolonged ICU stays. Furthermore, the marked tracheal dilatation results in clinical challenges with alveolar recruitment (44).

The management of tracheomegaly in the ICU remains largely supportive, as evidence-based therapeutic guidelines are lacking. Preventive strategies focus on minimizing tracheal injury by carefully monitoring cuff pressures—ideally maintaining them below 25 cmH_2_O whenever feasible—to avoid further mucosal damage (44). Regular assessment and documentation of cuff pressures, along with prompt attention to significant or persistent peritubal leaks are essential. In patients with tracheostomy, standard tracheostomy tubes are often inadequate, as their cuff may rest within the dilated segment of the trachea, failing to provide an effective seal. In such cases, adjustable-length tracheostomy tubes or extra-long tracheostomy devices are recommended to position the cuff below the area of maximal ectasia, thereby improving ventilatory efficacy and reducing the risk of additional tracheal injury. Adjunctive measures include bronchodilator therapy, mucolytics, targeted antibiotic treatment for infections, and chest physiotherapy to facilitate airway clearance. In selected cases, non-invasive ventilatory support such as CPAP may provide benefit by opening collapsible airway segments and improving expiratory flow limitation. While surgical interventions (e.g., tracheal stenting, tracheobronchoplasty) may be considered in refractory cases, these are associated with significant risks and are generally not recommended during the acute phase of critical illness.

### 6.3 Tracheal laceration

While tracheal lacerations can result from severe open or blunt cervical-thoracic trauma, iatrogenic causes—including tracheal intubation, tracheotomy, and rigid bronchoscopy—represent the most common etiologies. Post-intubation tracheal laceration is an extremely rare complication, occurring in approximately 0.005% of endotracheal intubations (45). Several mechanisms have been proposed to explain the development of tracheal lacerations, linked to a variety of risk factors that range from anatomical predispositions to purely mechanical factors occurring during or immediately after intubation. Predisposing anatomical factors include congenital tracheal anomalies, female sex, age over 50 years, short stature (< 165 cm), and structural airway weakening due to chronic inflammatory conditions such as COPD, tracheobronchomalacia, prolonged corticosteroid therapy, or the presence of lymphadenopathy, neoplasms, or mediastinal diseases (46). Most lesions involve the posterior wall of the trachea, namely the membranous part of the lower third, involved in 60%−80% of cases. Cardillo et al. (47) proposed a classification system for tracheal ruptures based on the extent and depth of the lesion: Level I: mucosal or submucosal injury without mediastinal emphysema or esophageal injury; Level II: complete tracheal lesion with mediastinal or subcutaneous emphysema, but without esophageal involvement or mediastinitis; Level IIIA: complete tracheal lesion with herniation of mediastinal tissue and esophagus into the tracheal lumen, but without mediastinitis or esophageal rupture, Level IIIB: Any rupture of the tracheal wall with esophageal injury or mediastinitis. Suspected tracheal rupture is primarily a clinical diagnosis, and a few patients are asymptomatic. The most frequent clinical sign (observed in 65%−80% of cases) is thoracic subcutaneous emphysema, which is also considered a protective feature, allowing early suspicion and a more rapid diagnostic work-up. Other clinical signs include dyspnea, cyanosis, hemoptysis (often minimal), pneumothorax (with positive pressure ventilation potentially causing tension pneumothorax), and persistent air leaks following chest drainage (48). Chest pain and hypotension (due to reduced cardiac filling caused by mediastinal shift) are less common. Acute mediastinitis is a rare complication of tracheal rupture, unlike esophageal rupture, where mediastinitis is almost invariably present. The interval between injury and symptoms onset can vary considerably, up to 72 h. Bronchoscopy remains the gold standard for definitive diagnosis, enabling direct visualization of the lesion, assessment of its exact location, extent, and depth, as well as the detection of any herniation of mediastinal structures. Moreover, bronchoscopy facilitates rapid therapeutic intervention, such as repositioning or re-intubating the patient, and monitoring the progression of conservative management. Bronchoscopy is often indicated even prior to imaging, based solely on clinical suspicion, given its superior sensitivity. Chest radiography may reveal signs of cervical or mediastinal emphysema, particularly along the spinal column. Pneumothorax may also be present but is missed in approximately 40% of cases and only subsequently identified on thoracic CT. Thoracic CT is highly sensitive for detecting cervical and mediastinal emphysema, pneumothorax, and pneumopericardium. It can identify signs of tracheal rupture and their location in approximately 71% of cases. With the use of multiplanar reconstructions, this sensitivity approaches 100% (49). There is no universally accepted consensus regarding the management of post-intubation tracheal laceration. Surgery has historically been the mainstay of treatment and continues to be employed in most cases. However, in recent years, conservative management has gained increasing acceptance, with selection criteria remaining a subject of debate. In general, patients must be hemodynamically and in stable respiratory conditions. The length and depth of the rupture, as well as the presence of mediastinitis, are key considerations in guiding therapeutic choice. According to several authors, surgical treatment is indicated for transmural lacerations longer than 2 cm, especially those in para-carinal location with esophageal wall prolapse into the tracheal lumen. Other indications for surgery include significant air leaks through pleural drainage, extensive pneumomediastinum, subcutaneous emphysema, or early signs of mediastinitis (50). Conversely, lesions shorter than 2 cm or not involving all the layers of tracheal wall, located within the cranial two-thirds of the trachea, or occurring in patients with high surgical risk, may be treated with conservative management. Non-surgical treatment in mechanically ventilated patients includes: endotracheal intubation (under bronchoscopic guidance), with the cuff positioned distal to the lesion, pleural drainage in the presence of pneumothorax, appropriate antibiotic coverage to minimize infection risk, administration of mucolytics and cough suppressants, regular bronchoscopic aspiration to prevent secretion build-up.

Following the therapeutic scheme proposed by Cardillo et al. (51) level I and level II injuries should generally be managed conservatively. Level IIIA injuries, although considered at high-risk, can also be treated non-surgically in centers with significant expertise in tracheal management. Level IIIB injuries invariably require surgical management. A case series by Cardillo et al. demonstrated that on 28 patients with Level I or II injuries managed conservatively, 27 healed successfully at 28-day follow-up. The choice of the surgical approach depends on the location and on the extent of the rupture (47). For lesions involving the proximal trachea and larynx, a cervicotomy or cervical mediastinotomy are preferred. A right posterolateral thoracotomy—the traditional approach—is recommended when the laceration extends to the membranous part of the main bronchi.

Endoscopic approaches have emerged as valuable adjuncts or alternatives to surgical repair in selected cases of tracheal laceration, particularly in patients with high surgical risk or in lesions of intermediate complexity where a conservative approach alone might be insufficient. Among the available endoscopic options, airway stenting and the application of fibrin glue have been increasingly reported in the literature. Airway stents—typically silicone-based and customizable—offer an effective means for bridging the tracheal defect, thereby promoting healing and maintaining airway patency. Silicone stents have the advantage of being removable and modifiable; fenestrations can be tailored to accommodate a concomitant tracheostomy tube when required. In patients with transmural lesions not suitable for immediate surgical repair, or when surgery is contraindicated, stenting can provide stabilization of the airway and prevent further complications such as progression of subcutaneous emphysema or pneumomediastinum. Clinical reports have documented favorable outcomes, with resolution of air leak and improved ventilation following stent placement, as well as successful subsequent stent removal once healing was confirmed (52, 53).

**Figure 6:**
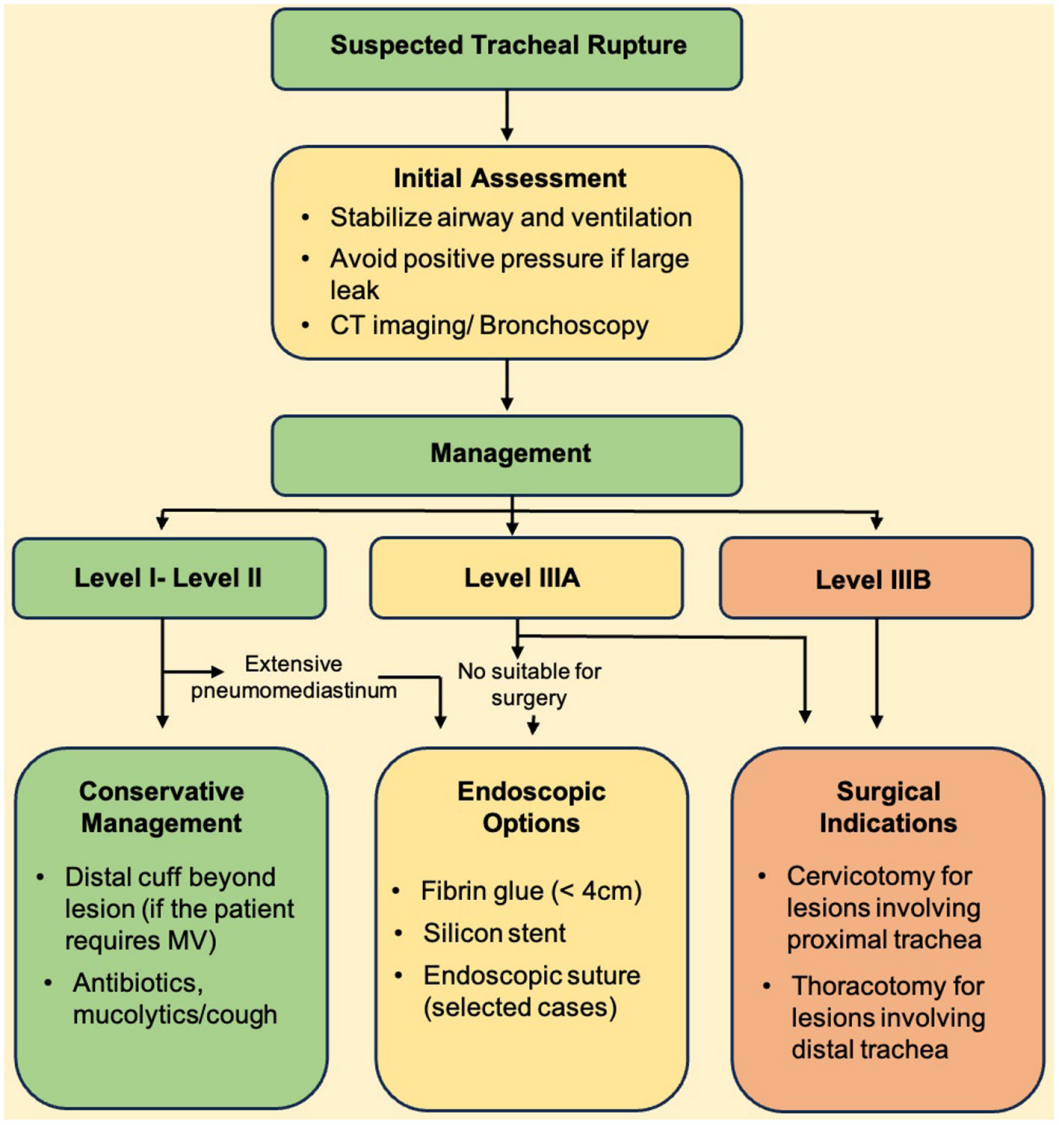
Decision-making algorithm in tracheal laceration.

Fibrin glue instillation represents another minimally invasive option, particularly suitable for small to moderate-sized lesions ( ≤ 4 cm) without esophageal injury, located in the upper or middle third of the trachea (54, 55). This technique involves bronchoscopic application of a two-component fibrin sealant directly into the laceration, leading to clot formation and tissue sealing. The method is generally performed under deep sedation with spontaneous breathing, avoiding the need for general anesthesia. In the series reported by Fiorelli et al., and Leonardi et al. the endoscopic application of fibrin glue resulted in successful closure of the tracheal defect in most patients, with durable healing confirmed at follow-up. Both techniques underscore the importance of careful patient selection. Fibrin glue is best suited for hemodynamically stable patients with superficial lesions and no evidence of infection or progressive emphysema. Stenting may be reserved for more complex scenarios, such as larger transmural lacerations, lesions associated with ventilatory failure, or when bridging support is needed to facilitate tissue healing. Overall, endoscopic treatments—including stenting and fibrin glue application—should be considered part of the multidisciplinary armamentarium for managing tracheal lacerations. Their judicious use, guided by lesion characteristics, patient conditions, and institutional expertise, may help to reduce morbidity associated with surgical repair while promoting safe and effective healing. Figure 6 illustrates the decision-making algorithm in tracheal laceration.

## 7 Tracheoesophageal fistula in critically ill patients

Tracheoesophageal fistula (TEF) is a rare but life-threatening condition, defined by an abnormal communication between the trachea and the esophagus. In critically ill patients, particularly those undergoing prolonged mechanical ventilation, benign TEFs most commonly result from ischemic necrosis caused by excessive pressure of endotracheal or tracheostomy tubes' cuff (56). Additional risk factors include patient agitation, nasogastric tube-induced exerting posterior pressure, respiratory or esophageal infections, steroids use, immunosuppression, and advanced age. In this setting, spontaneous healing is exceedingly rare. TEF should be suspected in intubated patients who present with persistent air leaks despite an inflated cuff, unexplained abdominal distension, recurrent pneumonia, or failure to wean from the ventilator (57). In awake patients, the so-called “Ono's sign”—coughing immediately after swallowing—is a classic but non-specific clinical indicator (58).

Diagnostic work-up typically includes computed tomography and combined bronchoscopy and esophagogastroduodenoscopy (EGD), which help in localizing and characterizing the fistula. Eesophagography with water-soluble contrast may also be useful but carries the risk of aspiration. In ventilated patients, flexible bronchoscopy is preferred and may reveal the fistula directly or through indirect signs, such as bubbling or presence of secretions coming from the digestive tract (59). Management strategies are highly individualized. Conservative management may include distal repositioning of the endotracheal tube cuff, maintenance of low cuff pressures (< 20 mmHg), and nutritional support via gastrostomy or jejunostomy (60). However, definitive treatment is often required to prevent recurrent aspiration, pneumonia, and respiratory failure. Surgical repair remains the definitive treatment for benign TEF, with excellent long-term outcomes when patients are optimized and weaned from mechanical ventilation. However, many ICU patients are not immediate surgical candidates due to their critical status. In such cases, interventional endoscopic techniques—most notably stenting—serve as vital bridging or palliative options (61). In both malignant and inoperable benign TEFs, esophageal stenting is generally considered the first-line endoscopic intervention, as it provides direct sealing of the fistula from the digestive side and often allows resumption of enteral feeding. Covered self-expanding metallic stents (SEMS) are preferred due to their sealing properties and resistance to migration. When esophageal stenting alone is insufficient—either due to persistent fistula or significant airway compromise—airway stenting or dual (esophageal and airway) stenting may be indicated. In the case of malignant TEFs, double stenting has shown to improve symptom control and survival (62). In a prospective study by Herth et al., 112 patients with malignant airway-esophageal fistulas underwent either airway, esophageal, or combined stenting. The subgroup receiving both airway and esophageal stents demonstrated a mean survival of approximately 253 days, with a significant improvement in quality of life (QoL) post-procedure. Importantly, the need for dual stenting was often determined intra-procedurally based on incomplete sealing of the fistula after single stent placement (63). Ultimately, in critically ill patients unfit for surgery, a tailored endoscopic approach combining stenting, infection control, and nutritional support remains the cornerstone of TEF management, with dual stenting offering an effective palliative option in selected cases. However, with the improvement of oncologic prognoses due to the advent of immunotherapy and targeted therapies, the durability of dual stenting may be challenged. Over time, the persistent radial pressure exerted by both stents on the shared tracheoesophageal wall can lead to progressive ischemia and mucosal necrosis. This mechanical trauma may promote fistula enlargement and limit the long-term effectiveness of the treatment, especially in patients with prolonged survival. Therefore, in selected cases, dual stenting should be carefully weighed against alternative or staged strategies. In carefully selected cases, autologous *fascia lata* grafts associated with double stenting have shown promise for fistula closure, although supporting evidence is still limited to case reports (64).

## 8 Impact of central airway diseases on extubation and weaning failure

Weaning from mechanical ventilation (MV) represents a pivotal phase in the management of critically ill patients, accounting for up to 40% of the total duration of ventilatory support (65). Despite its clinical relevance, the weaning process remains poorly standardized, and definitions of its onset vary widely (e.g., from the moment of intubation to sedation withdrawal, or the initiation of spontaneous breathing modes). Importantly, weaning duration is closely associated with patient outcomes, including survival, underscoring the need for structured approaches aimed at shortening this period. Evidence supports a multifaceted strategy including early transition to spontaneous breathing, minimization of sedation and neuromuscular blockade, and the use of weaning protocols or automated weaning systems.

Determining the optimal timing for liberation from MV remains a clinical challenge. Standardized screening criteria and the use of spontaneous breathing trials have been developed to guide clinicians, distancing them from a dogmatic view of gradual weaning (66). Among spontaneous breathing modalities, pressure support ventilation trials appear to offer advantages over traditional T-piece trials. Following extubation, noninvasive ventilation (NIV) has shown benefit in reducing reintubation rates, particularly in patients at high risk, such as those with COPD or congestive heart failure (67, 68).

Recent evidence also supports the use of high-flow nasal cannula oxygen therapy as an alternative to NIV in selected post-extubation settings (69).

Although most weaning difficulties are attributable to general factors such as respiratory muscle dysfunction, excessive sedation, or comorbid conditions, in rare cases central airways diseases [i.e., tracheal stenosis, malacia, or excessive dynamic airway collapse (EDAC)], may play a decisive role by limiting effective airflow or impairing the mechanics of spontaneous ventilation. Recognition and management of these conditions is essential, as they may require specific diagnostic and therapeutic interventions to achieve successful weaning.

### 8.1 Airway resistance and its role in weaning failure

The pathophysiology of weaning failure is complex and often multifactorial, typically involving a combination of airway and lung dysfunction, neurological dysfunction, cardiac dysfunction, diaphragmatic weakness, and endocrine disturbances. In this review, we focus primarily on airway-related factors, specifically analyzing how tracheal injuries and upper airway conditions may contribute to weaning failure.

Airway behavior during the weaning process is characterized by changes in airway resistance (70). Two principal components define airway resistance: flow resistance, and tissue resistance, the latter encompassing viscoelastic behavior and *Pendelluft* effects (i.e., redistribution of gas within the lungs without a net change in tidal volume). *Pendelluft* describes a gas movement from lung regions with low compliance (short time constants) to those with high resistance (long time constants), and is generally absent in healthy lungs (71). In normal lungs, tissue resistance is primarily due to viscoelastic dissipation within thoracic tissues. During controlled mechanical ventilation, airway resistance is typically reduced due to the positive pressure delivery, bronchodilation related to sedation, and the relative passivity of the respiratory muscles. Additionally, the caliber of the airways may increase due to higher tidal volumes, resulting in decreased flow resistance and reduced tissue resistance (72).

As the patient shifts to spontaneous breathing during weaning, total airway resistance tends to increase. This is due to several contributing factors, such as airflow reduction, airway caliber decrease, tissue resistance increased, airway inflammation and edema and small airway dysfunction. Airflow reduction: compared to controlled ventilation, spontaneous breathing is generally associated with lower inspiratory flow, which may increase resistive load in turbulent flow conditions. Decreased airway caliber: the loss of positive pressure support leads to a narrowing of the airways, particularly in the upper airway structures. This diameter reduction increases proximal flow resistance, particularly at the oropharyngeal and laryngeal levels (73). Increased tissue resistance: with the restoration of respiratory muscle activity, viscoelastic properties of the chest wall and lung parenchyma re-emerge as significant contributors to total resistance. This component may be further exacerbated by phenomena such as *Pendelluft*, in which asynchronous inflation of different lung regions leads to intrapulmonary gas redistribution (74). Airway inflammation and edema: post-extubation edema, especially in patients undergoing prolonged intubation or upper airway surgery, can lead to marked increases in upper airway resistance (75). Small airway dysfunction: in patients with underlying COPD or asthma, increased resistance of the small airways is a major determinant of weaning failure. Studies have shown that airway resistance significantly increases in COPD patients who fail spontaneous breathing trials (76). These changes in resistance impose a greater burden on the inspiratory muscles and may contribute to increased work of breathing, respiratory muscle fatigue, and eventual weaning failure. Furthermore, central airway diseases may worsen the changes in resistance during the weaning process, leading to waning failure. Recognition and targeted management of these conditions is therefore critical to improve weaning success.

## 9 Expiratory central airway collapse

Expiratory Central Airway Collapse (ECAC) is an umbrella term encompassing tracheobronchomalacia (TBM) and EDAC, both characterized by pathological narrowing of the central airways during expiration. While TBM reflects the degeneration of anterior cartilaginous support, EDAC is associated with weakness of the posterior membranous wall, which prolapses into the airway lumen during forced expiration. The clinical threshold for defining ECAC traditionally involves a >50% reduction in airway cross-sectional area during expiration compared to inspiration; however, a cutoff of ≥70% is more predictive of clinically significant disease and intraoperative airway compromise (77). The epidemiology of ECAC is confounded by heterogeneity in diagnostic criteria and a general lack of awareness among clinicians. Nevertheless, ECAC is frequently encountered in patients with chronic obstructive pulmonary disease (COPD), with prevalence estimates ranging from 13% to nearly 70% in selected populations (78). Contributing risk factors include advanced age, female sex, smoking, and prolonged corticosteroid use (79, 80).

Importantly, ECAC is underdiagnosed in the intensive care setting, where its presence may critically impair ventilatory mechanics and complicate liberation from mechanical ventilation. The pathogenesis of ECAC involves progressive weakening of the tracheobronchial wall, either anteriorly (TBM) or posteriorly (EDAC), resulting in dynamic collapse under positive pleural pressure during expiration. In COPD, chronic inflammation, tissue hypoxia, and repetitive pressure fluctuations contribute to chondrocyte degeneration and elastic fiber disruption. The resultant loss of airway integrity creates a functional expiratory flow limitation (EFL), localized anatomically at the EPP, beyond which transmural pressure becomes negative, promoting airway collapse (81). This phenomenon is particularly deleterious in mechanically ventilated patients and during spontaneous breathing trials (SBTs), where elevated inspiratory effort may exacerbate airway collapse, leading to air trapping, hypercapnia, and increased work of breathing. In the critical care setting, ECAC poses significant challenges to respiratory support. The transition from controlled ventilation to spontaneous modes may unmask or aggravate airway collapse, resulting in weaning failure. The increased negative pleural pressure during SBTs accentuates transmural gradients, which, in the context of weakened airway walls, predisposes to luminal occlusion. Clinically, this manifests as unexplained dyspnea, ineffective cough, secretion retention, oxygen desaturation, and frequent need for reintubation (82). These pathophysiological alterations may be erroneously attributed to COPD exacerbation or muscle weakness, delaying appropriate diagnosis. In addition, mechanical ventilation itself can further compromise airway integrity through prolonged cuff pressure-induced ischemia, particularly in patients with pre-existing vulnerability. Accurate diagnosis of ECAC requires a high index of suspicion and the integration of clinical, endoscopic, and radiological data. Flexible bronchoscopy remains the gold standard, enabling real-time assessment of airway dynamics under spontaneous breathing conditions. Characteristic findings include crescent-shaped, saber-sheath, or circumferential collapse patterns. Dynamic multidetector CT scans with inspiratory-expiratory phases offer a non-invasive alternative, with diagnostic accuracy comparable to bronchoscopy. Functional assessments, such as flow-volume loop analysis or impulse oscillometry, may support the diagnosis, though their specificity is limited (83). Management of ECAC in critically ill patients requires a tailored approach, balancing underlying disease burden, symptom severity, and procedural risks.

First-line therapy includes non-invasive positive pressure ventilation (NIPPV) modalities such as CPAP or BiPAP, which exert pneumatic stenting effects and may improve dyspnea and facilitate secretion clearance (84). Currently, airway pressure titration during dynamic bronchoscopy is considered a reliable method for determining the optimal level of positive pressure support. This procedure consists of performing a bronchoscopy while the patient performs tidal breathing and forced expiration maneuvers during CPAP (through the mask), allowing direct visualization of airway collapse. In a case report by Funes-Ferrada et al., CPAP was initiated at 6 cmH_2_O and progressively increased by 2 cmH_2_O until a reduction in airway collapse was observed. Stabilization of the airway during both tidal respiration and dynamic exhalation was achieved at a pressure of 8–10 cmH_2_O (85).

Several studies have also described the successful use of mechanical ventilation with PEEP ranging from 5 to 15 cmH_2_O in patients with EDAC, also during the weaning phase or under general anesthesia, to prevent respiratory complications (86, 87). Moreover, there is evidence supporting the use of high-flow nasal cannula oxygen therapy in patients who failed weaning from mechanical ventilation due to hypercapnia and impaired secretion clearance secondary to undiagnosed EDAC. In cases refractory to conservative measures, airway stenting serves both diagnostic and therapeutic purposes. Silicone Y-stents are preferred for short-term trials due to ease of removal, whereas metallic stents are reserved for non-surgical candidates with palliative intent. Tracheobronchoplasty (TBP) represents the definitive surgical option, entailing posterior splinting of the tracheobronchial wall with a mesh to restore airway patency. Although TBP is associated with improved symptom control and quality of life, it carries significant perioperative morbidity and is feasible only in highly selected patients (88, 89).

Laser (TBP) has emerged as a novel therapeutic option for patients with severe ECAC who are not suitable for surgical TBP. This technique induces fibrosis of the posterior membranous wall of the trachea, thereby limiting its excessive movement during expiration and reducing the degree of airway collapse (90). Despite its potential, laser TBP has not been widely adopted, largely due to concerns about the risk of damaging the posterior membranous wall, which is typically only 3–5 mm thick and particularly delicate. In mechanically ventilated patients, the presence of ECAC should be considered in the differential diagnosis of weaning failure, particularly when more common respiratory, neuromuscular, or cardiac causes have been excluded. Prompt recognition and targeted intervention can optimize ventilatory support strategies and prevent reintubation, morbidity, and prolonged ICU stay.

## 10 Obstructive fibrinous tracheal pseudomembrane (OFTP)

**Figure 7:**
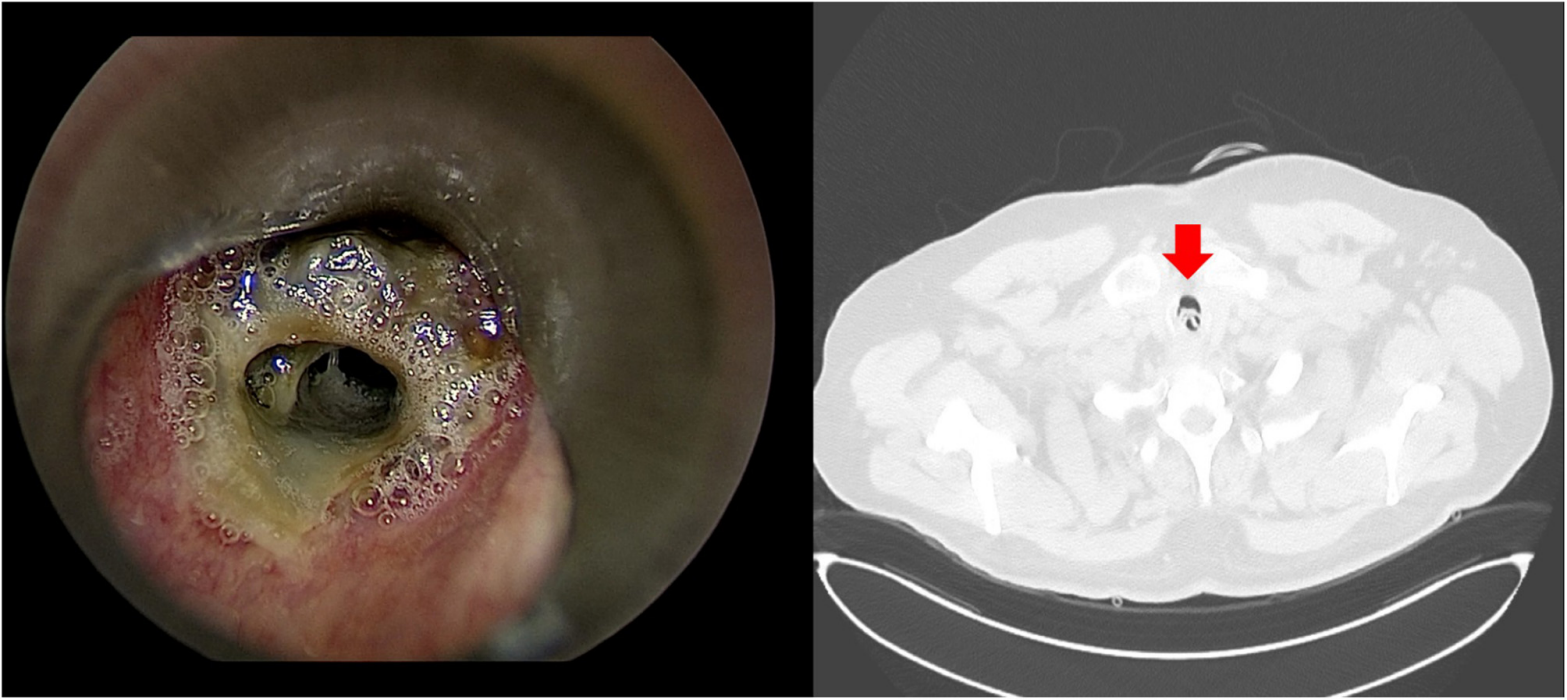
Bronchoscopic view and the axial CT scan of patient with obstructive fibrinous tracheal pseudomembrane.

Obstructive fibrinous tracheal pseudomembrane (OFTP) is a rare but potentially life-threatening complication following endotracheal intubation. It is characterized by the formation of a thick, tubular pseudomembrane adherent to the tracheal wall, typically at the site corresponding to the position of the endotracheal tube cuff. Depending on the degree of airway obstruction, OFTP can present with symptoms ranging from mild stridor to acute respiratory failure or even sudden death (91). On bronchoscopic examination, OFTP appears as a whitish or yellowish membrane partially or completely occluding the tracheal lumen, most frequently in the upper third of the trachea (Figure 7). Histology consists of fibrinous material with polymorphonuclear infiltration and areas of desquamated, often necrotic, tracheal epithelium (92). Several mechanisms have been proposed for the development of OFTP. The most widely accepted is ischemic injury of the tracheal mucosa and submucosa which can lead to mucosal necrosis, cartilage exposure, chondritis, and ultimately to pseudomembrane formation. Mucosal ischemia appears to occur predominantly in the region of the cricoid cartilage, which represents the most rigid and least distensible segment of the airway. Accordingly, it is believed that mucosal ischemia results primarily from direct compression of the endotracheal tube against the cricoid cartilage, rather than from the pressure exerted by the cuff itself. Even short-term intubation, traumatic airway instrumentation, or high-volume low-pressure cuffs maintained at safe pressures (< 25 cmH_2_O) have been associated with OFTP. Other contributing factors include systemic hypotension (e.g., during sepsis or cardiopulmonary resuscitation), microvascular compromise (e.g., in diabetes), aspiration of gastric contents, caustic inhalation, or coexisting inflammatory airway disease. A study on animals has demonstrated that aspirated gastric secretions can accumulate above the cuff and contribute to damaging the tracheal mucosa. Symptoms onset typically occurs within a few hours to days after extubation (median 24 h), though delays up to 2 weeks have been reported. Symptoms are often nonspecific and may include hoarseness, stridor, cough, or respiratory distress. OFTP is frequently misdiagnosed as glottic edema, laryngospasm, or secretion retention (93). Bronchoscopy remains the gold standard for diagnosis and reveals the extent and location of the obstruction. While chest radiography may show indirect signs (e.g., hyperinflation or atelectasis), computed tomography can sometimes identify the tracheal membrane, but it is not always diagnostic. Treatment consists in the removal of the pseudomembrane. Rigid bronchoscopy is often preferred in adults, particularly in cases with significant obstruction or respiratory distress, while flexible bronchoscopy is commonly used in children or stable adults. In most reported cases, bronchoscopic removal resulted in immediate clinical improvement, with very low rates of recurrence. A high index of suspicion is critical to avoid misdiagnosis and unnecessary reintubation. Early bronchoscopy remains the cornerstone for both diagnosis and definitive treatment (94). Figure 7 illustrates the bronchoscopic view and the CT scan of patient with Obstructive Fibrinous Tracheal Pseudomembrane.

## 11 Conclusion

Central airway diseases, though relatively uncommon, represent a critical and often underrecognized cause of respiratory failure in the intensive care setting. Their clinical manifestations may mimic more common conditions such as asthma and COPD exacerbations, or post-extubation complications, leading to diagnostic delay and suboptimal management. Bronchoscopy remains the cornerstone of diagnosis and treatment, offering both direct visualization and therapeutic options in a single procedure. A high index of suspicion, prompt endoscopic evaluation, and a multidisciplinary approach are essential to improve outcomes. Recognition of central airway involvement should be an integral part of the diagnostic algorithm in ICU patients with unexplained weaning failure, persistent respiratory distress, or atypical ventilatory patterns. Timely identification and appropriate intervention can significantly reduce morbidity and may be lifesaving.

Looking ahead, however, several important knowledge gaps and unmet clinical needs remain. Addressing these will require better epidemiologic data, refinement of diagnostic tools, preventive strategies against iatrogenic injuries, and the development of innovative therapies. These issues are outlined in the following section on Future Directions.

## 12 Future directions

Despite significant progress in understanding and managing central airway diseases in the ICU, important gaps remain.

First, robust epidemiological studies are needed to define the true prevalence of central airway pathology among critically ill patients, since most current data come from small case series or single-center experiences. Such knowledge would allow intensivists to better stratify risk and integrate airway assessment into standard ICU protocols.

Second, outcomes research should focus not only on short-term survival but also on long-term morbidity, quality of life, and functional status after ICU discharge. Identifying prognostic markers may help clinicians tailor management strategies and prioritize interventions in patients most likely to benefit.

Third, preventive strategies warrant greater emphasis. Advances in endotracheal tube design, automated cuff-pressure monitoring, and standardized airway care bundles could reduce the incidence of iatrogenic injuries. Education and training in safe airway management should remain a priority for ICU teams.

Finally, innovative therapies are under development. Biodegradable stents, bioengineered tracheal scaffolds, and regenerative medicine approaches hold promise for selected patients, though clinical translation is still limited. Future research should also explore less invasive, targeted interventions for expiratory central airway collapse and fibrotic airway stenosis, with the aim of reducing dependence on surgery or permanent stents.

A stronger focus on these areas, coupled with multidisciplinary collaboration, will help shape the next generation of strategies to prevent, diagnose, and treat central airway diseases in the critical care environment.
